# Unrevealing Lithium Repositioning in the Hallmarks of Cancer: Effects of Lithium Salts (LiCl and Li_2_CO_3_) in an In Vitro Cervical Cancer Model

**DOI:** 10.3390/molecules29184476

**Published:** 2024-09-20

**Authors:** Juan Carlos García-Acosta, Alejando Israel Castillo-Montoya, Gareth Omar Rostro-Alonso, Edgar Yebrán Villegas-Vázquez, Laura Itzel Quintas-Granados, Luis Sánchez-Sánchez, Hugo López-Muñóz, Lizbeth Cariño-Calvo, Israel López-Reyes, Lilia Patricia Bustamante-Montes, Gerardo Leyva-Gómez, Hernán Cortés, Nadia Judith Jacobo-Herrera, Rosario García-Aguilar, Octavio Daniel Reyes-Hernández, Gabriela Figueroa-González

**Affiliations:** 1 Laboratorio de Farmacogenética, UMIEZ, Facultad de Estudios Superiores Zaragoza, Universidad Nacional Autónoma de México, Ciudad de México 09230, Mexico; jcarlosga99@gmail.com (J.C.G.-A.); alex24castillomontoy@gmail.com (A.I.C.-M.); rostroalonsogarethomar@gmail.com (G.O.R.-A.); eyebran.villegas@gmail.com (E.Y.V.-V.); octavio.reyes@zaragoza.unam.mx (O.D.R.-H.); 2 Colegio de Ciencias y Humanidades, Plantel Cuautepec, Universidad Autónoma de la Ciudad de México, Ciudad de México 07160, Mexico; itzel.quintas@uacm.edu.mx (L.I.Q.-G.); israel.lopez.reyes@uacm.edu.mx (I.L.-R.); 3 Laboratorio de Biología Molecular del Cáncer, UMIEZ, Facultad de Estudios Superiores Zaragoza, Universidad Nacional Autónoma de México, Ciudad de México 09230, Mexico; luisss@unam.mx (L.S.-S.); hugolm@comunidad.unam.mx (H.L.-M.); 4 Facultad de Ciencias Químicas, Universidad Veracruzana, Orizaba 94340, Mexico; lcarino@uv.mx; 5 Coordinación de Investigación, Centro Universitario Siglo XXI, Edo. de México 03100, Mexico; liliapatricia.bustamante@cus21.edu.mx; 6 Departamento de Farmacia, Facultad de Química, Universidad Nacional Autónoma de México, Ciudad de México 04510, Mexico; leyva@quimica.unam.mx; 7 Centro de Investigación y de Estudios Avanzados del Instituto Politécnico Nacional (CINVESTAV-IPN), Zacatenco, Ciudad de México 07360, Mexico; 8 Laboratorio de Medicina Genómica, Departamento de Genómica, Instituto Nacional de Rehabilitación Luis Guillermo Ibarra, Ciudad de México 14389, Mexico; hcortes_c@hotmail.com; 9 Unidad de Bioquímica, Instituto Nacional de Ciencias Médicas y Nutrición Salvador Zubiran, Ciudad de México 14080, Mexico; nadia.jacoboh@incmnsz.mx; 10 Laboratorio de Citometría de Flujo y Hematología, Diagnóstico Molecular de Leucemias y Terapia Celular (DILETEC), Ciudad de México 07800, Mexico; rgarcia@diletec.com.mx

**Keywords:** lithium chloride, lithium carbonate, cervical cancer cells, apoptosis induction, cell cycle arrest, migration inhibition

## Abstract

Lithium, a natural element, has been employed as a mental stabilizer in psychiatric treatments; however, some reports indicate it has an anticancer effect, prompting the consideration of repurposing lithium for cancer treatment. The potential anticancer use of lithium may depend on its form (salt type) and the type of cancer cells targeted. Little is known about the effects of Li_2_CO_3_ or LiCl on cancer cells, so we focused on exploring their effects on proliferation, apoptosis, migration, and cell cycle as part of the hallmarks of cancer. Firstly, we established the IC_50_ values on HeLa, SiHa, and HaCaT cells with LiCl and Li_2_CO_3_ and determined by crystal violet that cell proliferation was time-dependent in the three cell lines (IC_50_ values for LiCl were 23.43 mM for SiHa, 23.14 mM for HeLa, and 15.10 mM for HaCaT cells, while the IC_50_ values for Li_2_CO_3_ were 20.57 mM for SiHa, 11.52 mM for HeLa, and 10.52 mM for HaCaT cells.) Our findings indicate that Li_2_CO_3_ and LiCl induce DNA fragmentation and caspase-independent apoptosis, as shown by TUNEL, Western Blot, and Annexin V/IP assay by flow cytometry. Also, cell cycle analysis showed that LiCl and Li_2_CO_3_ arrested the cervical cancer cells at the G1 phase. Moreover, lithium salts displayed an anti-migratory effect on the three cell lines observed by the wound-healing assay. All these findings imply the viable anticancer effect of lithium salts by targeting several of the hallmarks of cancer.

## 1. Introduction

Cervical cancer (CC) is the fourth most common malignancy in women. In 2022, a total of 662,301 new cases were reported by the International Agency for Research on Cancer, causing 348,874 deaths [1]. Low-income and middle-income countries reported 90% of CC cases mainly due to the lack of screening and vaccination initiatives. In contrast, in high-income nations, CC rates and fatalities have decreased by over 50% in the last three decades following the implementation of structured screening programs [2,3,4]. Most of the CC cases arise from infection with the Human Papillomavirus (HPV), with HPV DNA detected in about 95% of cervical lesions [5]. 

Most HPV infections are transient and disappear naturally. In some cases, persistent infection may lead to premalignant conditions such as cervical intraepithelial neoplasia or adenocarcinoma in situ. In most women, without treatment, progression from dysplasia to invasive carcinoma may take several years to decades. However, in approximately 10% of cases, this progression can occur in less than a year [6]. Diagnosis through Papanicolaou testing is challenging when adenocarcinoma in situ appears, which is believed to contribute to the rising incidence of this specific subtype of CC [7].

Cervical cancer treatment is based on the guidelines of the International Federation of Gynecology and Obstetrics (FIGO) [8]. Treatment options depend on the stage of the disease at diagnosis and local resources. It generally includes cone biopsy, radical trachelectomy, pelvic lymph node dissection, radical hysterectomy, pelvic radiotherapy, brachytherapy, chemoradiation, or a combination thereof [8,9,10]. However, these treatment options have disadvantages such as the narrow application scope, drug resistance, acute and long-term side effects, damage to proliferating healthy tissues, structural deformities, systemic toxicity, and psychological problems [11,12]. 

Lithium is a natural element used as a mental stabilizer of bipolar disorders since the 20th century and to help against suicide [13,14]. Evidence in vitro and in vivo proposes lithium salts for cancer treatment while reducing side effects [15,16]. Recently, our research group conducted a review on lithium as an antitumor agent [17]. Several investigations revealed that the salt LiCl increased apoptosis in lymph node carcinoma of the prostate cells [18], decreased the proliferation of ameloblastoma cells [19], and, in human multiple myeloma cell lines, inhibited cell proliferation and induced cell cycle arrest [20]. In addition, LiCl reduced the proliferation and colony formation capabilities of human head and neck squamous cell carcinoma [21] and improved the efficacy of chemotherapeutic agents by enhancing non-apoptotic cell death in a colorectal carcinoma model [22]. When LiCl is combined with temozolomide, it induces cell death via NFAT1/FasL signaling in human glioblastoma cells [23] and, combined with mitomycin C, induces autophagy in breast cancer cells [24]. Moreover, LiCl sensitized colon cancer cells to radiation therapy [25], inhibited GSK-3*β*, and decreased the expression of markers of the mesenchymal phenotype in triple-negative breast cancer lines [26]. Lithium citrate (Li_3_C_6_H_5_O_7_) induced apoptosis in hepatocarcinoma in vitro [27]. Lithium carbonate (Li_2_CO_3_) induced apoptosis and autophagic cell death in HCC-29 cells [27] and arrested the cell cycle in the G2/M phase in the HCC-29 cell line [28]. Li_2_CO_3_ also inhibited cell proliferation and stimulated cell death in skin melanoma cells through induction of autophagy and apoptosis [29]. Although there is enough evidence of the effect of lithium salts on different in vitro cancer models, the effect of lithium on cervical cancer is lacking. This evidence has opened up the possibility of repurposing lithium for the treatment of cancer. In this research, we conducted experiments using two lithium salts (Li_2_CO_3_ and LiCl) on two cervical cancer cell lines (HeLa and SiHa) and on a non-tumoral cell line (HaCaT) to investigate the effects of lithium on proliferation, DNA fragmentation, migration, and cell cycle arrest. 

Together, our results suggest that both lithium salts affected cervical cell proliferation in a time-dependent manner and induced DNA fragmentation in CC cell lines. Moreover, both salts induced the basal expression of PARP-1 and proCAS-3 as proteins associated with apoptotic cell death biomarkers. Finally, LiCl and Li_2_CO_3_ arrested the cell cycle of HeLa, SiHa, and HaCaT cells at the G1 phase and inhibited cell migration. Our findings give light to the potential anticancer effect of lithium salts by targeting the hallmarks of cancer, specifically focusing on cell proliferation, pro-apoptotic, anti-migratory, and cell cycle arrest mechanisms.

## 2. Results

### 2.1. Effect of Lithium Salts over Cell Proliferation of CC

The crystal violet assay measures cell viability and indirectly determines cell proliferation in groups of cells treated with substances that promote cell death. Adherent cells are detected by crystal violet stain that binds to proteins and DNA. Dead cells lose their adherence and are subtracted from the cell population, reducing the violet stain in the culture [30]. 

**Figure 1:**
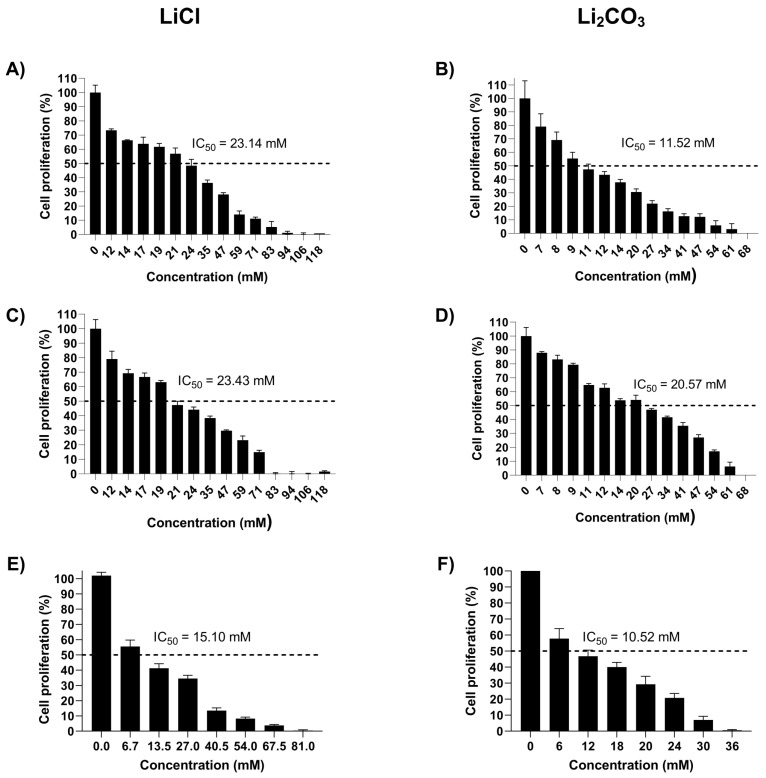
Effect of lithium salts over Cell Proliferation of CC. The proliferation of Hela (**A**,**B**), SiHa (**C**,**D**), and HaCaT (**E**,**F**) cells treated with different concentrations of LiCl or Li_2_CO_3_ for 24 h was assessed by crystal violet staining assay. Values represent the mean ± S.D. and are expressed as a percentage of the control cells. Three independent biological replicate measurements with technical triplicates were performed.

Thus, the cell proliferation percentage at 24 h of exposure to several LiCl and Li_2_CO_3_ concentrations was obtained (Figure 1). Both lithium salts induce cell proliferation in a concentration-dependent manner in HeLa (Figure 1A,B), SiHa (Figure 1C,D), and HaCaT (Figure 1E,F) cells. Table 1 shows the IC_50_ values that depend on the genotype of cells and the lithium salts. The IC_50_ values calculated for LiCl are in the same range for SiHa and HeLa cell lines. In contrast, for HeLa cells, the IC_50_ values for Li_2_CO_3_ are different, and the IC_50_ from SiHa is 1.79-fold higher than that of HeLa cells. For the non-tumorigenic cell line (HaCaT) the IC_50_ values were lower than tumorigenic cell lines. Considering these data, the IC_50_ values were selected for further analysis. 

### 2.2. Effect of Lithium Salts over the DNA Fragmentation of CC Cell Lines

#### 2.2.1. Effect of Lithium Salts over the Apoptotic Protein’s Expression of CC Cell Lines

To further clarify the effect of lithium on apoptosis, we detected by Western Blot the levels of biomarkers of the execution phase of apoptosis: CAS-3 and PARP-1. CAS-3 in its active form weighs 17 kDa, and sometimes only its precursor form can be detected: proCAS-3 (36 kDa). PARP-1 is used to determine if there is proteolytic activity of CAS-3. When there is apoptosis, the active form of PARP-1 (110 kDa) is cleaved by caspases, generating an 89 kDa fragment. If PARP-1 (110 kDa) increases, it indicates that a response to genetic damage is activated [31,32]. 

**Figure 2:**
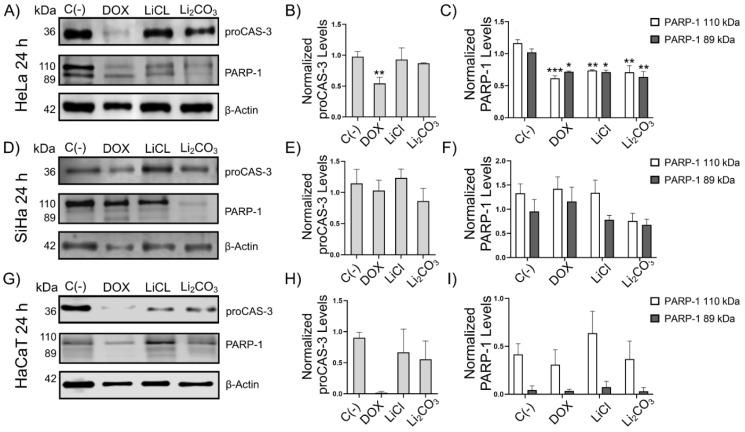
Effect of lithium salts over the expression of apoptotic biomarker proteins at 24 h of stimuli. Western Blot analysis to detect PARP-1 in HeLa (Panels **A**–**C**), SiHa (Panels **D**–**F**), and HaCaT (Panels **G**–**I**) cells untreated [C(-)] or after 24 h of LiCl or Li_2_CO_3_ treatments. PARP-1 was also detected in cells treated with Dox. Two bands corresponding to full-length PARP-1 (110 kDa) and cleaved isoform (89 kDa) were detected in the samples. *β*-actin was detected as a loading control. All experiments were assessed in triplicate and the values are expressed as the mean ± SD with a (* *p* < 0.05, ** *p* < 0.002, *** *p* < 0.001). *β*-actin was used as a loading control in all samples.

**Figure 3:**
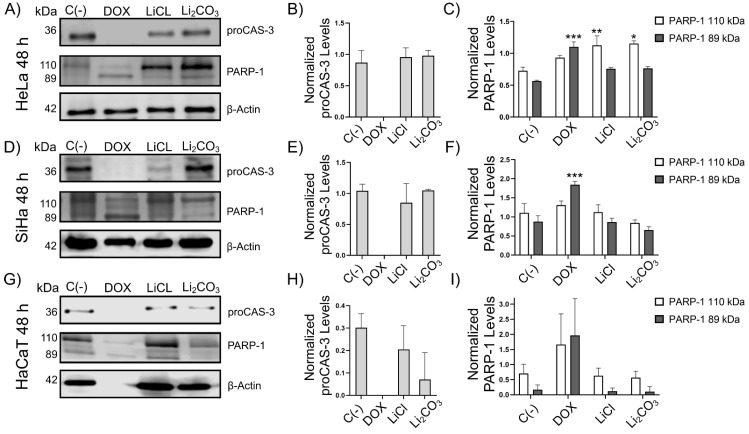
Effect of lithium salts over the expression of apoptotic biomarker proteins at 48 h. Western blot analysis to detect PARP-1 in HeLa (Panels **A**–**C**), SiHa (Panels **D**–**F**), and HaCaT (Panels **G**–**I**) cells untreated [C(-)] or after 48 h of LiCl or Li_2_CO_3_ treatments. PARP-1 was also detected in cells treated with Dox. Two bands corresponding to full-length PARP-1 (110 kDa) and cleaved isoform (89 kDa) were detected in the samples. *β*-actin was detected as a loading control. All experiments were assessed in triplicate and the values are expressed as the mean ± SD with a * *p* < 0.05, ** *p* < 0.002, *** *p* < 0.001.

In all cases, proCAS-3 had no significance between cells, groups, and time. Full-length PARP-1 and cleaved-PARP-1 expressions were observed in SiHa, HeLa, and HaCaT cells at 24 h (Figure 2) and 48 h (Figure 3). The expression of PARP-1 was slightly increased after LiCl or Li_2_CO_3_ treatment compared to untreated cells or cells treated with Dox at 48 h of stimuli. Statistical analysis of normalized PARP-1 expression levels in HeLa, SiHa, and HaCaT showed a difference significantly according to one-way ANOVA used to compare between groups (* *p* < 0.05). Interestingly, in LiCl-treated cells, the expression of PARP-1 is higher than Li_2_CO_3_-treated cells (* *p* < 0.05, ** *p* < 0.002, *** *p* < 0.001). *β*-actin was used as a loading control in all samples. It is worth mentioning that in HaCaT cells treated with Doxorubicin (10 µM) for 48 h, *β*-actin protein was not detected since Doxorubicin is a powerful cell death agent that causes the degradation of cytoskeleton components; this does not compromise the Western Blot results since it demonstrates the effect of different stimuli on the tumor cell phenotypes of greatest interest [33]. The accumulation of the active form of PARP-1 and its 89 kDa fragment varied depending on the cell line and the stimulation time, where in HeLa, there was a significant reduction at 24 h, while, at 48 h, the active form of PARP-1 had a considerable increase. In SiHa, there was only a trend in the reduction of PARP-1 levels with Li_2_CO_3_, and in HaCaT there were no significant differences. This shows that lithium salt activity is only in HeLa during the execution phase.

#### 2.2.2. TUNEL Assay of LiCl- and Li_2_CO_3_-Treated Cells

**Figure 4:**
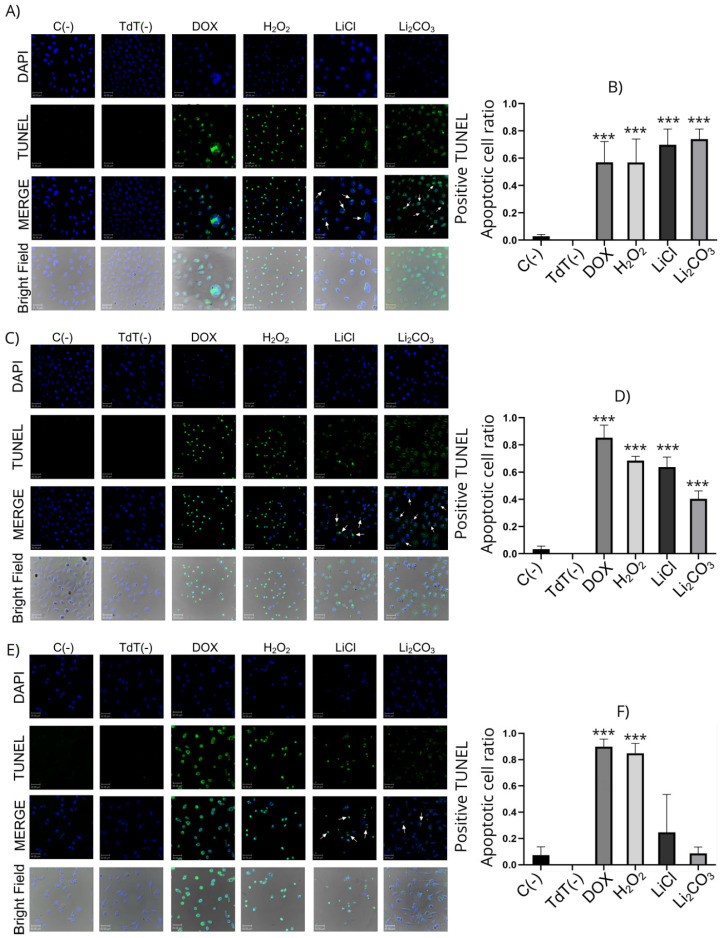
Fluorescent TUNEL labeling of lithium-treated HeLa cells (panels **A**,**B**), SiHa (panels **C**,**D**), and HaCaT (panels **E**,**F**). Cells were treated with LiCl or Li_2_CO_3_ for 24 h before TUNEL labeling—images for TUNEL staining overlay green fluorescence from the TUNEL stain with blue fluorescent from DAPI. Positive apoptotic cells exhibit the characteristic fragmented nuclei (arrows). More apoptotic cells appeared in the presence of Li_2_CO_3_. Untreated cells [C(-)] or cells tested in the absence of TdT enzyme [TdT(-)] or cells treated with Dox or H_2_O_2_ were included as controls. Magnification ×400, scale bar represents 40 µm. We observed a statistically significant difference between LiCl and Li_2_CO_3_ treatments. All experiments were assessed in triplicate, and the values are expressed as the Mean ± SD with *** *p* < 0.001.

**Figure 5:**
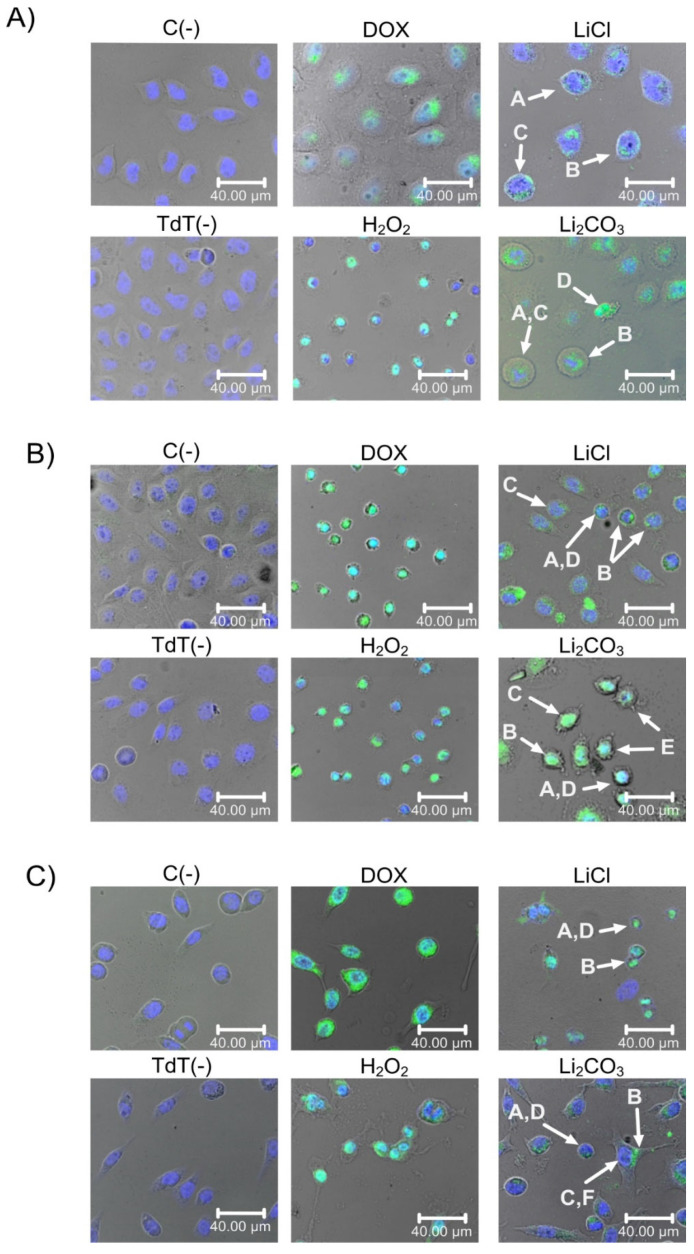
Effects of lithium salts on the morphology of HeLa (panel **A**), SiHa (panel **B**) and HaCaT (Panel **C**) cells. Untreated cells [C(-)], cells tested in the absence of TdT enzyme [TdT(-)], cells treated with Dox or H_2_O_2_ were included as controls. Arrows point the apoptotic cells in lithium salt treatment groups. Morphology parameters used for the cell count and analysis are pointed with arrows, where (A) shows nuclear condensation and round morphology acquisition, (B) DNA fragmentation seen with TUNEL positive staining within the nucleus, (C) membrane integrity maintenance, (D) Cell shrinkage, (E) membrane blebbing and (F) cell with no morphology change but with DNA fragmentation. Scale bar 40.00 µM.

The TUNEL assay was performed to detect the 3′-OH ends generated by DNA fragmentation due to apoptosis by activating endonucleases through enzymatic labeling with nucleotides conjugated with FITC. Altogether, DAPI staining is performed to detect DNA and locate the nuclei, evaluate their morphology and nuclear integrity, and determine if the TUNEL signal corresponds to the location of the genetic material within the cells. Thus, this experiment was assessed to determine the lithium-induced DNA fragmentation (Figure 4 and Figure 5) [32,34]. 

After 24 h of treatment, DNA fragmentation was observed in the lithium-treated cells. In SiHa and HeLa cells, the mean fluorescence intensity after lithium treatment increased compared to untreated cells [C(-) and TdT(-)]. The percentages of positive TUNEL apoptotic cell ratios were 69.8%, 63.8%, and 24.7% for HeLa, SiHa, and HaCaT cells treated with LiCl, respectively. On the other hand, for Li_2_CO_3_ treatment, the ratios of total TUNEL-positive cells were 73.9%, 40.4%, and 8.7% for HeLa, SiHa, and HaCaT cells, respectively. 

A morphology analysis showed in Figure 5 was performed for the cell count determination in bright fields and for DAPI/TUNEL tinction. Apoptosis morphology characteristics are seen in HeLa, SiHa, and HaCaT cells treated with LiCl and Li_2_CO_3_, where cell round morphology and nuclear condensation were observed. DNA fragmentation marked by TUNEL-positive staining within the nucleus, cell integrity, and membrane blebbing were also analyzed. Other types of observed cells maintained the cell line morphology and still showed TUNEL-positive staining.

#### 2.2.3. Flow Cytometry Apoptosis Assay (Annexin V/IP)

The annexin V/PI assay allows for determining the viability and processes of cell death due to apoptosis and necrosis of cells by evaluating the integrity and polarity of their membrane. Viable cells do not present Annexin V/PI staining; necrotic cells present staining with only PI, while cells in early apoptosis only present staining with Annexin V, and late apoptosis staining with both markers is present. Annexin V staining indicates the translocation of phosphatidyl serine, a phenomenon that occurs during apoptosis [35]. 

**Figure 6:**
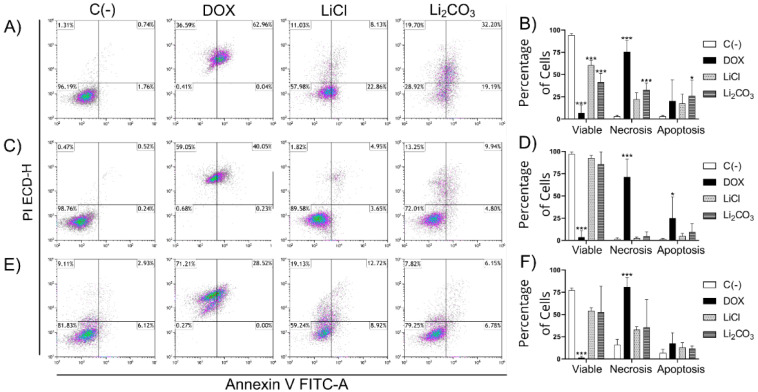
Quantification of cell apoptosis by flow cytometry on HeLa cells (panels **A**,**B**), SiHa cells (panels **C**,**D**), and HaCaT (panels **E**,**F**). The upper left quadrant means Annexin V-negative, PI-positive (Annexin V−, PI+) cells, defined as necrotic cells. The upper right quadrant means Annexin V-positive, PI-positive (Annexin V+, PI+) cells, defined as late apoptotic cells. The lower left quadrant means Annexin V-negative, PI-negative (Annexin V−, PI−) cells, which are defined as viable cells. The lower right quadrant means Annexin V-positive, PI-negative (Annexin V+, PI−) cells, which are defined as early apoptotic cells. Panels (**B**,**D**,**F**) are shown in the statistical analysis when comparing the lithium-treated with the control groups. All experiments were assessed in triplicate, and the values are expressed as the mean ± SD with * *p* < 0.033 and *** *p* < 0.001.

As shown in Figure 6, cells treated with lithium displayed apoptosis compared to the control group. Nevertheless, the striking results focus on Li_2_CO_3_ in HeLa Cells (panels A and B), which exhibit more apoptosis and less necrosis than the positive control. It is interesting that lithium salts do not show high necrosis levels against cervical cells but do show apoptosis at different levels, favoring HeLa cells. 

### 2.3. Effect of Lithium Salts on Cellular Migration of CC Cell Lines (Wound-Healing Assay) 

**Figure 7:**
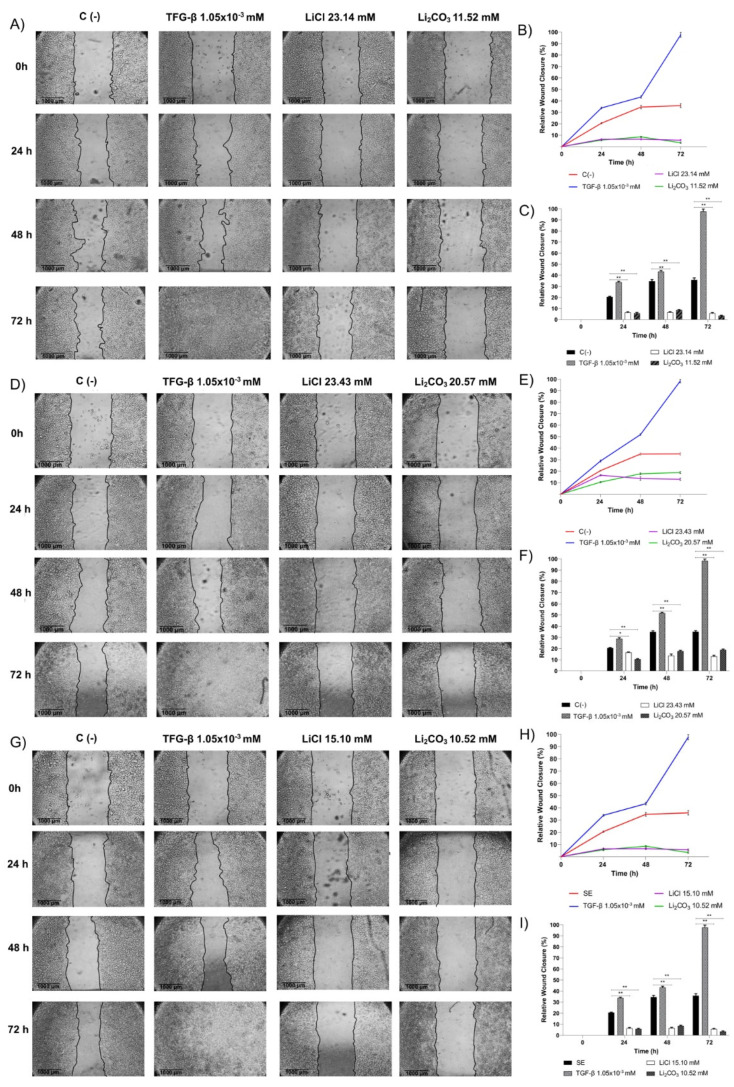
Effect of lithium salts over cellular migration of HeLa, SiHa, and HaCaT cells. Panel (**A**,**D**,**G**) Cells were treated with LiCl (23.14 mM), Li_2_CO_3_ (11.52 mM), and TGF-*β* (105 mM), or untreated cells [C(-)]. Photographs were taken of the gaps observed under a microscope at different time points, i.e., 0 h, 24 h, 48 h, and 72 h. The lines delineate the migrating edges of cells. Panels (**B**,**E**,**H**) Relative migration for all tested conditions. Panels (**C**,**F**,**I**) Relative migration percentages were calculated for all tested conditions. Bars represent the mean of three different measurements, and the S.D. is indicated in each bar. A statistical analysis was performed comparing tested conditions with the negative control (* *p* < 0.01, ** *p*< 0.001).

Wound-healing assays were conducted to investigate whether LiCl or Li_2_CO_3_ can influence cell migration in CC cells (Figure 7) since metastasis is known to be a big challenge for cancer patients, and these experiments currently represent a solid basis for state-of-the-art research focused on understanding metastatic potential and the development of possible targeted anti-metastatic therapies [36]. According to Bouchalova et al., 2022 [36], processes such as migration and cell invasion capacity are evaluated for the study of metastasis in in vitro models. The wound assay evaluates basic cell migration parameters by forming a monolayer and generating a wound. The ability of the cells to fill the wound is measured using software image J to determine the level of cell migration.

Cell migration of SiHa, HeLa, and HaCaT cell lines was significantly enhanced after TGF-*β* treatment (positive control) compared to untreated cells [negative control, C(-)]. Conversely, in Hela (Figure 7, Panels A–C), SiHa (Figure 7, Panels D–F), and HaCaT (Figure 7, Panels G–I) cells, the presence of lithium salts significantly reduced cell migration evaluated at 24, 48, and 72 h (** *p* > 0.001). In SiHa cells, the relative migration was 16.52% at 24 h and 13.34% at 48–72 h after LiCl treatment, while for Li_2_CO_3_ treatment, the relative migration was 10.51% at 24 h to 18.29% at 48–72 h. In HeLa cells, relative migration was 6.44% for 24, 48, and 72 h after LiCl treatment and 5.75%, 8.58%, and 5% for 24, 48, and 72 h after Li_2_CO_3_ treatment, respectively. In HaCaT cells, relative migration was 6.21% for 24, 48, and 72 h after LiCl treatment, and for Li_2_CO_3_ treatment, the relative migration was 5.73%, 6.54%, and 3.4% for 24, 48, and 72 h, respectively. In negative controls [C(-)] for SiHa, HeLa, and HaCaT cells, the relative migration was 20.51% for 24 h and 35.02% for 48–72 h, while in positive controls (TGF-*β*), the relative migration was 28.95–34.5% for 24 h, 42.31–52.31% for 48 h and 97–98% for 72 h.

For the statistical data, a one-way ANOVA test was performed, followed by a Tukey test. All experiments were assessed in triplicate, and the values are expressed as the Mean ± SD with * *p* < 0.01 and ** *p* < 0.001 compared to the control group with the LiCl and Li_2_CO*_3_* treatments.

### 2.4. Effect of Lithium Salts over the Cell Cycle Progression of CC Cell Lines

The amount of DNA in cells varies depending on the phase of the cell cycle, and by staining with PI, which binds proportionally to the amount of DNA within a cell, the phase of the cycle in which it occurs can be analyzed. In this experiment, we used asynchronous cultures, which are essential for conducting a comprehensive cell cycle analysis, allowing for a general overview of cell behavior across all cell cycle stages under normal growth conditions. In a tumor, cells proliferate asynchronously, simultaneously occupying various cell cycle phases. Thus, an asynchronous culture replicates this dynamic, providing a more realistic model to evaluate cellular responses to anticancer treatments [37]. 

**Figure 8:**
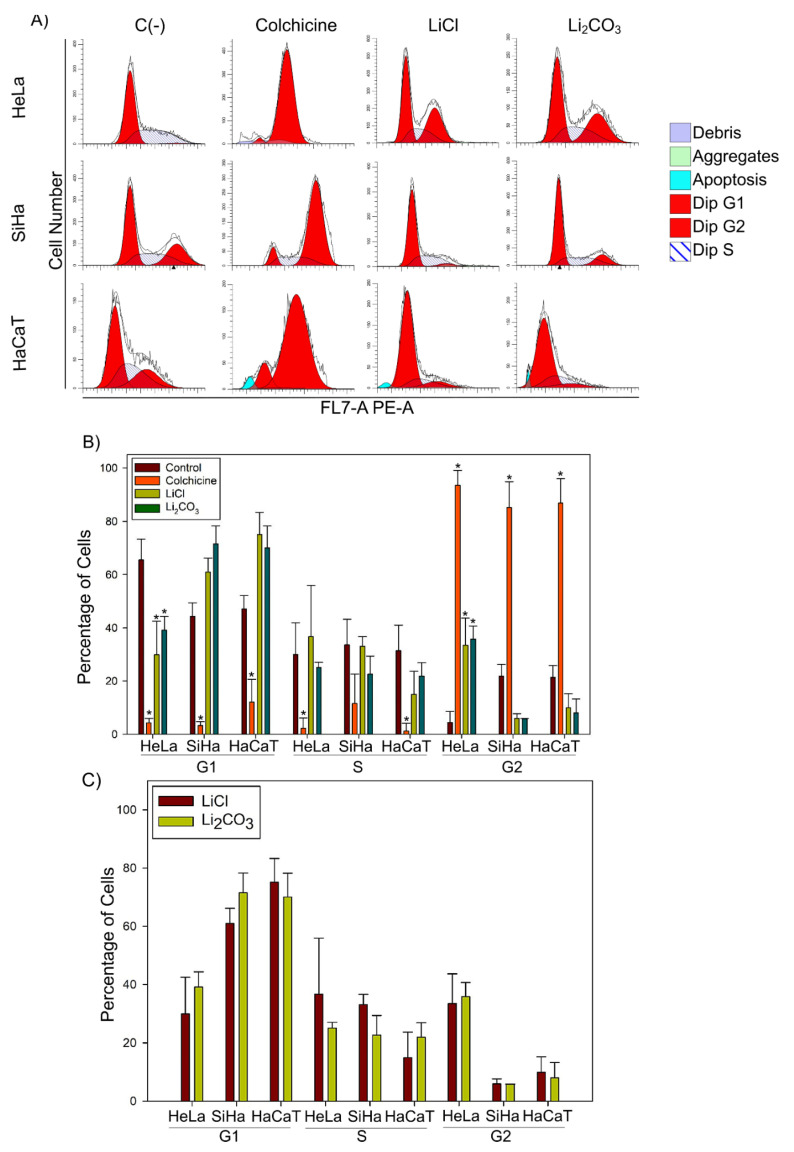
Effect of lithium salts over the cell cycle of CC cell lines: (**A**) Representative flow cytometry graphs of cell cycle distribution of SiHa, HeLa, and HaCaT cell lines untreated [C(-)] and treated with colchicine (0.0247 mM), LiCl (23.43 mM for SiHa, 23.14 mM for HeLa, and 15.10 mM for HaCaT cells) and Li_2_CO_3_ (20.57 mM for SiHa, 11.52 mM for HeLa, and 10.52 mM for HaCaT cells) (**B**) Cell percentages of treated and untreated SiHa, HeLa, and HaCaT cells in G1, S, and G2 phases of cell cycle. Bars indicate the mean of three independent biological replicate measurements with technical triplicates and the SD of each bar is indicated and a * *p* < 0.033 obtained from one-way ANOVA analysis. (**C**) Comparison between cell lines. Statistical analysis suggests no difference between SiHa and HeLa cells treated with LiCl or Li_2_CO_3_.

To determine the effect of lithium salt over the cell cycle progression, we analyzed the cell cycle distribution of SiHa, HeLa, and HaCaT cells treated with LiCl or Li_2_CO_3_ (Figure 8). 

The proportion of HeLa cells in the G1 phase was 29.89% ± 12.61% with LiCl treatment and 39.11% ± 5.24% with Li_2_CO_3_ treatment, in contrast to 65.53% ± 7.87% for untreated cells. For the S phase, HeLa cell percentages were 36.66% ± 19.57% with LiCl and 25.06% ± 1.98% with Li_2_CO_3_, compared to 30.01% ± 11.90% for untreated cells. Following lithium treatments, there was an increase in the proportion of HeLa cells in the G2 phase. Specifically, 33.45% ± 10.21% of HeLa cells treated with LiCl and 35.83% ± 4.86% with Li_2_CO_3_ accumulated in the G2 phase, whereas only 4.47% ± 4.05% of untreated cells accumulated in this phase (Figure 8, panel A). 

The percentage of SiHa cells in the G1 phase was 60.96% ± 5.23% for LiCl treatment and 71.54% ± 6.73% for Li_2_CO_3_ treatment, compared to 44.36% ± 5.01% of untreated cells. The percentage of SiHa cells in the S phase for LiCl and Li_2_CO_3_ treatments was 33.08% ± 3.53% and 22.62% ± 6.77%, respectively, and 33.59% ± 9.86% for untreated cells. The percentage of SiHa cells in the G2 phase was dramatically decreased after lithium treatments. We found that 5.97% ± 1.72% of SiHa cells treated with LiCl or 5.85% ± 0.04% of SiHa cells treated with Li_2_CO_3_ accumulated in the G2 phase, while untreated cells that accumulated in the G2 phase were 21.92% ± 4.36% (Figure 8, panel A).

An ANOVA analysis of cells at the G1 and G2 phase showed that LiCl or Li_2_CO_3_ treatments had a statistically significant difference with control (untreated cells) and with cells treated with colchicine (positive control) (*p* < 0.05) (Figure 8, panel B). For HeLa cells, we found a statistically significant difference between cells in the G1 phase after LiCl or Li_2_CO_3_ treatment compared to untreated SiHa cells, suggesting that LiCl or Li_2_CO_3_ treatment inhibited the progression of the cell cycle at the G1 phase. According to statistical analysis by one-way ANOVA, we found a statistically significant difference between cells in the G1 phase after LiCl or Li_2_CO_3_ treatment compared to colchicine-treated SiHa cells. No statistical differences were observed for cells accumulating in the S phase in SiHa or HeLa cell lines. Evidence from the HeLa cell line suggested that LiCl arrested the cell cycle at the S phase, whereas Li_2_CO_3_ arrested the cell cycle at the G1 phase. On the other hand, SiHa cells treated with LiCl or Li_2_CO_3_ arrested the cell cycle at the G1 phase. The non-tumoral cell line (HaCaT) cell cycle was arrested at the G1 phase after LiCl or Li_2_CO_3_ treatments.

To determine if there is a difference between CC cell lines and the lithium treatment, we compared HeLa and SiHa cells treated with LiCl or Li_2_CO_3_. We found that cells distributed differently compared to cell lines. When cells were exposed to LiCl, SiHa cells exhibited a G1 phase accumulation of 60.96% ± 5.23%, whereas HeLa cells showed a lower accumulation of 29.89% ± 12.61%. Similarly, treatment with Li_2_CO_3_ resulted in 71.54% ± 6.73% of SiHa cells accumulating in G1, compared to only 39.11% ± 5.24% of HeLa cells (Figure 8, Panel C). In contrast, no statistically significant difference was found when LiCl treatment was compared with Li_2_CO_3_ treatment in HeLa or SiHa cells (Figure 8, panel C).

## 3. Discussion

Cancer treatment encounters several challenges, including drug resistance, metastasis, exacerbated proliferation, and disease relapse. Thus, this work focuses on lithium salts as a new alternative for targeting some of the hallmarks of cancer, exhorting its repurposing as anticancer agents. Previous studies point out that lithium has antitumor properties over several cancer types, such as prostate [18], ameloblastoma [19], multiple myeloma [20], human head and neck squamous cell carcinoma [21], hepatocarcinoma [27], and skin melanoma [29]. However, there is a lack of evidence of the potential antitumoral effect of different lithium salts in cervical cancer. We provide data showing that the type of lithium salt acts differently depending on the CC genotype.

Cell proliferation of SiHa, HeLa, and HaCaT cells decreased along with the increase in LiCl or Li_2_CO_3_ concentrations, suggesting that lithium salts inhibited cell proliferation in a concentration-dependent manner.

Although the IC_50_ values of LiCl are similar for SiHa and HeLa cells, when treated with Li_2_CO_3_, the IC_50_ value for SiHa cell line was twice that for HeLa cells, signifying that lithium’s nature may be relevant to the repositioning of this drug in addition to low toxicity and high bioavailability of organic lithium salts [38]. Earlier publications indicate that 50 and 100 mM LiCl inhibited the tumor cell growth rate and activated the Wnt/*β*-catenin signaling pathway in the human CC CaSki cell line [39], supporting the statement of the difference in activity of lithium salts among genotypes.

The effects of lithium on the execution phase of apoptosis were mostly seen in HeLa cells, where alterations in PARP-1 are observed at 24 and 48 hours. The decrease in PARP-1 forms (110 and 89 kDa) at 24 h would indicate that there could be proCAS-3 or some other protease activity and that DNA repair mechanisms are being inhibited. The increase in the active form of PARP-1 at 48 hours in Hela suggests the activation of the damage response due to the occurring DNA fragmentation. In SiHa, on the other hand, the low activity of lithium salts on PARP-1 and proCAS-3 and the high viability on annexin V/PI, together with the presence of morphological changes and DNA fragmentation that were observed, suggests that there could be a programmed cell death independent of caspases, where characteristics of apoptosis with lower kinetics are observed. In HaCaT, the apoptotic effects of lithium salts on PARP-1 and proCAS-3 are consistent with the results in the other experiments, where a low killing effect on viability and induction of DNA fragmentation is observed. Thus, induction of apoptosis is evident except when analyzing the pro-apoptotic proteins by WB (PARP-1 and proCAS-3). This could mean that these cells’ programmed cell-death process is caspase-independent. A similar phenomenon was described in the neuroblastoma cell line B65, where lithium treatments increased cell death by apoptosis without activating CAS-3 activity. Even with caspase inhibitors, apoptosis still occurred, indicating a caspase-independent pathway [40]. Similarly, Karlovic and colleagues (2007) found that LiCl treatments (20 mmol/L) in A1235 glioblastoma cells altered the levels of proteins such as Bcl-2 and proCAS-3, and there was no cleavage of PARP1 [40], reinforcing with our work that lithium does not affect this pathway.

**Figure 9:**
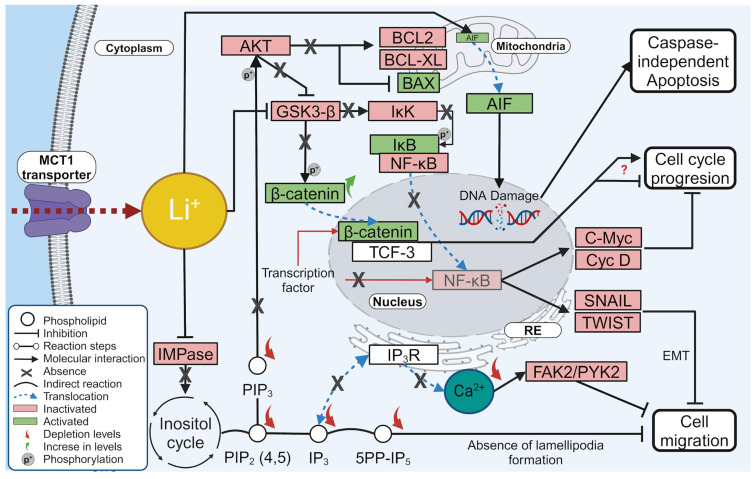
Proposal mechanism of lithium salts effect over in cervical cancer cells model.

In addition, the translocation of phosphatidylserine from the inner side of the cell membrane to the outer side is associated with apoptosis. This translocation typically occurs due to caspase activation. On the other hand, we evaluated apoptosis by flow cytometry with the Annexin V/PI assay using HeLa cells treated and untreated with lithium salts, where the externalization of phosphatidylserine does occur, indicating cell death by apoptosis. In SiHa cells treated with lithium salts, this effect is observed to a lesser extent, with greater cell viability. Still, the lithium salts exhibited significantly less necrosis in all cases than the reference positive control drug. We also observed DNA fragmentation in the TUNEL assay by LiCl in SiHa (63.8%), HeLa (69.8%), and HaCaT (24.7%) cell lines, while for Li_2_CO_3_, the DNA fragmentation was for SiHa (40.4%), HeLa (73.9%), and HaCaT (8.7%). Moreover, Figure 5 shows morphological changes related to apoptosis. The integrity of the membrane, cell shrinkage and round morphology, blebbing, nuclear condensation, and DNA fragmentation were considered for the cell count. Thus, it is demonstrated that lithium salts induce DNA fragmentation and morphological apoptotic alterations in HeLa, SiHa, and, to a lesser extent, HaCaT cells. DNA fragmentation is the main feature of apoptosis caused by the caspase-activated DNA fragmentation factor (DFF). However, other caspase-independent DNA fragmentation factors could be activated, as ENDO-G and AIF (allows other endonuclease mechanisms to be activated) [41], suggesting that Lithium induces a caspase-independent cell death (CICD) by these factors (Figure 9). According to Tait, S.W.G., & Green, D.R. (2008) [42], in CICD, there is slower kinetics compared to normal apoptosis, so this would explain why, despite there being fragmentation and morphological changes, viability remains high in the annexin V/PI assay. 

To study the anti-metastatic potential of lithium salts over cervical tumor cells we assessed an anti-migratory assay (wound-healing assay), since migration is the initial step of the complex metastatic process [36]. Both lithium salts had an anti-migratory effect on SiHa, HeLa, and HaCaT cells. LiCl inhibited SiHa, HeLa, and HaCaT migration in 86.66%, 93.56%, and 93.79%, respectively. On the other hand, Li_2_CO_3_ inhibited 81.71%, 95%, and 96.6% of the migration of SiHa, HeLa, and HaCaT, respectively. The percentage of the inhibition of migration was 10.5%-fold higher for HeLa cells compared to SiHa at 24, 48, and 72 h. No differences were observed between LiCl and Li_2_CO_3_ treatments regarding the anti-migratory effect.

Our results suggested that LiCl and Li_2_CO_3_ inhibited the migration and cell proliferation of SiHa and HeLa cell lines more selectively than HaCaT cells used as a non-tumoral genotype. Although the specific mechanism of the inhibitory effect of LiCl and Li_2_CO_3_ is unknown, we propose, in Figure 9, the mechanistic actions of lithium salts in the functional experiments conducted in this work. However, further molecular evidence is needed to demonstrate these hypotheses.

Based on the literature review and Figure 9, lithium might inhibit both GSK-3*β* and IMPase by entering the cell via MCT1; it reduces PIP2, PIP3, and IP3 levels, affecting AKT activation and calcium release from the endoplasmic reticulum, and possibly inhibiting cell migration [43]. Additionally, IP3 is a precursor of the 5PP-IP5 isoform, whose depleted levels may also be associated with the inhibition of cell migration due to the lack of lamellipodia formation [44].

Finally, the observed cell cycle arrest in the G1 phase may be caused by preventing the activation of the NF-κB pathway, which affects the transcription of genes involved in the cell cycle [45].

For instance, when lithium inhibits GSK-3*β*, it prevents the phosphorylation of *β*-catenin, causing its accumulation and translocation to the nucleus along with TCF-3, resulting in an anti-proliferative effect by inhibiting DNA synthesis [46]. Simultaneously, GSK-3*β* is a positive regulator of the NF-κB pathway. When GSK-3*β* is active, it regulates the IKK protein, which is responsible for phosphorylating IκB, leading to its degradation and the release of NF-κB. This release allows NF-κB to translocate to the nucleus and target various genes, including EMT activation (SNAIL and TWIST) and cell cycle regulation (C-Myc and Cyclin D). Therefore, lithium’s inhibition of GSK-3*β* would inhibit this signaling pathway and all its downstream targets, favoring the inhibition of cell migration and cell cycle arrest [47].

## 4. Materials and Methods

### 4.1. Cell Culture

Human cervical cancer HeLa (infected with HPV-18), SiHa (infected with HPV-16), and HaCaT (non-tumorigenic cell line) cell lines were cultured in RPMI medium (LABORATORIOS MICROLAB S.A. DE C.V., catalog number: M-221P) containing 10% heat-inactivated neonatal calf serum (In vitro, catalog number S-02) and antibiotic–antimycotic (In vitro, catalog number A-07) and incubated at 37 °C and 5% CO_2_ in an incubator (NUARE, Brossard, QC, Canada, NU-8600). When the cell reached 80–90% confluence, the culture was treated with 0.25% versene (Sigma-Aldrich; Milwaukee, WI, USA) and centrifuged (6000× *g*) at 4 °C for 10 min. Pellet cells were counted using a hemocytometer and immediately used in the assays.

### 4.2. Reactive and Materials

LiCl (423.9 mg, Sigma Aldrich; Milwaukee, WI, USA) and Li_2_CO_3_ (442.4 mg, Sigma Aldrich; Milwaukee, WI, USA) were dissolved in 50 mL fresh sterile medium and filtrated (2 µm) to obtain a stock solution of each salt: 200 mM for LiCl and 120mM for Li_2_CO_3_. Sterile lithium salts were added to the culture medium at the desired final concentrations for each experiment.

Doxorubicin (136 µL, Zuclodox, Zurich Pharma; Tepeji del Río, Mexico) was dissolved in 50 mL fresh sterile medium and filtrated (2 µm) to obtain a solution of 10 µM. At this concentration, the stimuli were added directly to the cells.

H_2_O_2_ (1.27 µL, Alcomex; Mexico City, Mexico) was added to 1 mL of fresh sterile medium and filtrated (2 µm) to obtain a final concentration of 125 µM H_2_O_2_ directly into the cell culture.

Cytarabine (10 mg, Santa Cruz Biotechnology; Dallas, TX, USA) was dissolved in H_2_O Mili Q sterile and filtrated (2 µm) to obtain a stock solution 510.78 µM. Sterile Cytarabine (ara-C) was added to the stock solution at 10 µM.

TGF-*β* (0.21 µM, Sigma Aldrich) was prepared as a work solution at 1.05 nM added in each well.

### 4.3. Proliferation Assay

Cells (7 × 10^3^ cells/well) were seeded in a 96-well plate for 24 h at 37 °C in a 5% CO_2_ incubator for the incubation period. Then, the treated-with-HeLa cells were treated with LiCl at different concentrations (0, 12, 14, 17, 19, 21, 24, 35, 47, 59, 71, 83, 94, 106, and 118 mM) and Li_2_CO_3_ (0, 7, 8, 9, 11, 12, 14, 20, 27, 34, 41, 47, 54, 61, and 68 mM), SiHa treated with at different concentrations LiCl (0, 12, 14, 17, 19, 21, 24, 35, 47, 59, 71, 83, 94, 106, and 118 mM) and Li_2_CO_3_ (0, 7, 8, 9, 11, 12, 14, 20, 27, 34, 41, 47, 54, 61, and 68 mM), and HaCaT with LiCl at different concentrations (0, 6.7, 13.5, 27, 40.5, 54, 67.5, and 81 mM) and Li_2_CO_3_ (0, 6, 12, 18, 20, 24, 30, and 36 mM). Times of incubation were 24 h. At the end of the incubation period, cells were washed with PBS pH 7.0, and 50 µL of 0.5% crystal violet staining solution was added and kept at room temperature for 20 m. Finally, the optical density of each well was determined at 570 nm with the Bio Tek EPOCH Microplate Spectrophotometer (Agilent; Santa Clara, CA, USA). The percentage of cytotoxicity was reported considering non-stimulated cells as control and expressed as the mean of three independent biological replicate measurements with technical triplicates that were performed. Data were analyzed with the Graphpad PRISM software (version 9.0.2) [48], to determine the IC_50_ value of each lithium salt for HeLa, SiHa, and HaCaT cells.

### 4.4. Determination of IC_50_

The mean IC_50_ values are the concentrations of lithium salts needed to inhibit cell viability by 50%, and these values were calculated using the graphical method. Cellular cytotoxicity (%) was plotted on the Y-axis directly against the lithium salt concentration on the X-axis. By extrapolation using the Graphpad PRISM software (version 9.0.2) [48], we determined the IC_50_ value of each lithium salt for HeLa, SiHa, and HaCaT cells.

### 4.5. Western Blot Assay (Apoptosis)

HeLa, SiHa, and HaCaT cells treated and untreated with lithium salts and Dox were lysed with RIPA buffer (Santa Cruz Biotechnology, Inc., sc-24948) containing 0.1% protease inhibitors (Complete Protease Inhibitor Cocktail, Roche, catalog number 11697498008) and incubated at 4 °C for 30 min, following which samples were centrifugated at 13,000× *g* at 4 °C for 25 min. Soluble protein concentrations of cell lysates were determined spectrophotometrically at 280 nm using the Bio Tek EPOCH Microplate Spectrophotometer (Agilent, Santa Clara, CA, USA). Samples with equal amounts of protein were mixed with 5X Laemmli sample buffer (10% SDS, 50% glycerol, 0.02% bromophenol blue, and 0.3125 M Tris HCl, pH 6.8.) supplemented with *β*-mercaptoethanol and boiled for 10 min. Then, 50 µg protein of each sample was separated into 8% and 12% SDS-PAGE gels for visualization of PARP-1 and Caspase-3 (Cas-3) proteins, respectively, in a vertical electrophoresis system (Mini Trans-Blot^®^ Cell, Bio-Rad, catalog number 1703810; Hercules, CA, USA). Gels were transferred onto 0.45 µm polyvinylidene difluoride (PVDF) membranes (Thermo Scientific, catalog number 88518; Rockford, IL, USA) in a mini Transblot^®^-Cell module (Bio-Rad; Hercules, CA, USA) at constant 400 mA for 2 h. Then, membranes were blocked with 5% non-fat milk in TBS pH 7.0 containing 0.1% Tween-20 for 2 h at room temperature. Then, membranes were incubated with antibodies against PARP-1 Rabbit pAb (1:3000 dilution, ABclonal, catalog number A0942; Woburn, MA, USA), Caspase-3 mouse mAb (1:1000 dilution, Santa Cruz Biotechnology, Inc., sc-7272; Dallas, TX, USA), and *β*-actin mouse mAb (1:10,000 dilution, ABclonal, catalog number AC004; Woburn, MA, USA) for 18 h at 4 °C on a rocking platform. After incubation period, membranes were washed five times with TBS pH 7.0 containing 0.1% Tween-20 and incubated for 1 h at 25 °C with a peroxidase-conjugated secondary antibodies anti-rabbit IgG (1:10,000 dilution, Cell Signaling Biotechnology, catalog number 7074s; Danvers, MA, USA) for PARP-1 detection or anti-mouse IgG (1:10,000 dilution, Cell Signaling Biotechnology, catalog number 7076s; Danvers, MA, USA) for Cas-3 and *β*-actin detection. Finally, membranes were washed with TBS pH 7.0 containing 0.1% Tween-20 and developed by chemiluminescence using Clarity MaxTM Western ECL (Bio-Rad, catalog number 1705062; Hercules, USA) following the instructions from the manufacturer. Finally, image analysis was performed using the C-DiGit Blot (LI-COR; Lincoln, IL, USA). All experiments were performed in triplicate. In all samples, *β*-actin was detected as a loading control and used to normalize target protein detection for densitometric analysis using the Image J software (version 7). Data normalization was performed by dividing densitometry data from the target protein with densitometry data from the loading control protein (*β*-actin). 

### 4.6. TUNEL Assay (Apoptosis)

HeLa, SiHa, and HaCaT cells (3 × 10^4^ cells/well) were seeded in 6-well plates with built-in glass coverslips and incubated for 24 h at 37 °C in a 5% CO_2_ incubator. Then, HeLa cells were treated with 23.14 mM LiCl and 11.52 mM Li_2_CO_3_; SiHa cells were treated with 23.43 mM LiCl and 20.57 mM Li_2_CO_3_; and HaCaT cells were treated with 15.10 mM LiCl and 10.52 mM Li_2_CO_3_, for 24 h. All cell cultures were treated as controls with 125 µM H_2_O_2_ or 10 µM Doxorubicin (Zuclodox, Zurich Pharma; Tepeji del Río, Mexico). Cell cultures were incubated for 4 days, and the culture medium was replaced every other day. At the end of the treatment, coverslips containing a monolayer of HeLa, SiHa, or HaCaT cells were fixed in 4% paraformaldehyde/0.1 M PBS (pH 7.0) at room temperature for 30 min and washed with PBS. Then, cells were stained with the In Situ Cell Death Detection Kit following the manufacturer’s instructions (Cat. No. 11 684 795 910, Roche; Manheim, Germany). Negative control cells with medium alone (C(-)) and without addition of TdT enzyme (TdT(-)) were included. All Cells were counterstained with DAPI (1:500 in PBS; Calbiochem; Gibbstown, NJ, USA) and all conditions were analyzed under a confocal microscope TC SP8 (DMI8, Leica, Wetzlar, Germany), with an excitation wavelength of 450–500 nm and a detection wavelength of 490–600 nm. All photos were taken with the same conditions of a 40× objective and had the 40 µm scale bar in the lower left corner, indicating homogeneity. Images were analyzed in LAS X Office software (version 1.4.5.27713, Leica microsystems; version 1.4.5.27713) to determine the fragmentation of cellular DNA, a hallmark of apoptosis.

### 4.7. Flow Cytometry (Annexin V/IP)

HeLa, SiHa, and HaCaT cells (5 × 10^5^ cells/well) were seeded in 6-well plates and after 24 h of growing the cells were stimulated with the IC_50_ of lithium salts: HeLa cells were treated with 23.14 mM LiCl, 11.52 mM Li_2_CO_3_, SiHa cells were treated with 23.43 mM LiCl, 20.57 mM Li_2_CO_3_, and HaCaT cells were treated with 15.10 mM LiCl, 10.52 mM Li_2_CO_3_ for 24 h. After that time, according to the manufacturer, the Annexin V/IP assay (FITC Annexin V Apoptosis Detection Kit II, No. 556547, BD BIOSCIENCES; San Diego, CA, USA) was assessed. Finally, the lectures were taken in Beckman Coulter Cytoflex S and data were analyzed in the Kaluza C versión 1.1 software.

### 4.8. Wound-Healing Assay

A wound-healing assay was performed to evaluate cell migration. In sterile conditions, an adhesive tape (0.5 mm width) was placed in 96-well plates, and a 2 mm division mark was made in the center of the wells to delimit the area for photographic analysis. Then, HeLa, SiHa, and HaCaT cells were seeded (3 × 10^4^ cells/well) in 100 µL of RPMI medium supplemented with 10% neonatal serum and incubated for 24 h at 37 °C with 5% CO_2_. At the end of the incubation period, an inhibitor of proliferation 10 µM cytarabine (ara-C) was added and incubated for 2 h at the same conditions described above. Subsequently, the tape was removed to form the wound. Samples were gently washed with PBS pH 7.0 and 23.14 mM and 23.43 mM LiCl, and 11.52 mM and 20.57 mM Li_2_CO_3_ were added to HeLa and SiHa cells, respectively. HaCaT cells were incubated with 15.10 mM LiCl and 10.52 mM Li_2_CO_3_. Negative control (untreated cells) and cells treated with 0.105 µM TGF-*β* were included in each experiment as a positive migration control. Cells were monitored at several incubations (0, 24, 48, and 72 h) and documented using a Canon EOS Rebel T100 camera. Three biological replicates with technical triplicates were performed for each measurement. Wound thickness was analyzed by bioinformatics tools (Image J) using images taken at different times in the predetermined area and quantifying the migration rate of the cells. The migration percentage (*Wound Closure* %) was calculated using the following formula:

$$Wound\;Closure\;\%=\left(\frac{A_{t=0}-A_{t=∆t}}{A_{t=0}}\right)\times 100\%$$

where *A_t_*_=0_ is the initial wound area, and *A_t_*_=Δ*t*_ is the wound area after n hours of the initial scratch. Relative migration was obtained in μm.

### 4.9. Flow Cytometry (Cell Cycle Analysis)

Cells (3 × 10^5^) were plated in a 6-well plate and incubated at 24 h at 37 °C with 5% CO_2_. Then, cells were untreated and treated with 20 µL colchicine (1 mg/mL, as a positive control) and lithium. HeLa cells were treated with 23.14 mM LiCl and 11.52 mM Li_2_CO_3_, whereas SiHa cells were treated with 23.43 mM LiCl and 20.57 mM Li_2_CO_3_, and HaCaT cells were treated with 15.10 mM LiCl and 10.52 mM Li_2_CO_3_. Cells were grown for 24 h at 37 °C with 5% CO_2_. After trypsinization, cells were collected by centrifugation at 1000× *g* (1500 rpm) for 3 min at room temperature. Then, cells were fixed in ice-cold methanol (500 µL) with PBS pH 7.0 (500 µL) at 4 °C for 1h. Cells were washed thrice in PBS pH 7.0 (1 mL) and centrifuged. Then, cells were treated with 30 µL RNase A (100 U/mL) and incubated for 30 min at 37 °C. Samples were centrifuged and resuspended in 500 µL of PBS with 5 µL propidium iodide (20 µg/mL) and analyzed using the Beckman Coulter Cytoflex S and data were analyzed in the ModFit Lt software (https://www.vsh.com/products/mflt/, accessed on 18 September 2024). We chose the colchicine concentration based on reported values known to arrest the cell cycle at the G2 phase. Three biological replicates with technical duplicates were performed for each measurement.

### 4.10. Statistical Analysis

All data were expressed as mean ± standard error of the mean (SEM). Differences between experimental groups were compared using ANOVA or *t*-student test using GraphPad 9 software. A confidence interval of 95% and a *p* = 0.05 for statistical significance were considered.

## 5. Conclusions

In summary, our research indicates that the cell proliferation of SiHa, HeLa, and HaCaT cells decreased along with the increase in LiCl or Li_2_CO_3_ concentrations, suggesting that lithium salts inhibited cell proliferation in a concentration-dependent manner; the IC_50_ values for Li_2_CO_3_ are lower than those obtained for LiCl, showing more sensitivity to the first salt. The data suggest that lithium salts trigger DNA fragmentation in cervical cancer cells, with Li_2_CO_3_ exhibiting a more pronounced effect. Additionally, LiCl and Li_2_CO_3_ induce apoptosis in a caspase-independent pathway. Finally, both lithium salts inhibit cell migration, leading to the cell cycle arrest in the G1 phase. Altogether, along with the toxicity safety compiled over time for other diseases, it supports further investigations of the lithium salts as anticancer agents, with a promising intervention in cell death, proliferation, and metastasis inhibition.

## Figures and Tables

**Table 1:** IC_50_ values of lithium salts for CC cell lines.

Cell Line	IC_50_ Values (mM)	
	LiCl	Li_2_CO_3_
HeLa	23.14 ± 1.344	11.52 ± 1.062
SiHa	23.43 ± 1.370	20.57 ± 1.313
HaCaT	15.10 ± 1.19	10.52 ± 1.009

## Data Availability

The original contributions presented in the study are included in the article, further inquiries can be directed to the corresponding author.
